# Supplementary material for: Antibiotic Resistance and Genetic Profiles of Vibrio parahaemolyticus Isolated from Farmed Pacific White Shrimp (Litopenaeus vannamei) in Ningde Regions
Source: Microorganisms. 2024 Jan 12;12(1):152. doi: 10.3390/microorganisms12010152 (PMC10821069; doi:10.3390/microorganisms12010152)
Supplement: Supplementary file 1 [file microorganisms-12-00152-s001.zip › Table S1.pdf]

Table S1. Criteria for determining the diameter of antibiotics zone of inhibition (mm).

Category	Disc content	Antibiotics	Susceptible	Intermediate	Resistant
Tetracyclines	30 μg	Tetracycline(TET)	≥19	15~18	≤14
	30 μg	Doxycycline(DOX)	≥16	13~15	≤12
Chloramphenicols (Amphenicol)	30μg	Flufenicol(FFC)	≥18	13~17	≤12
	30μg	Chloramphenicol(CHL)	≥18	13~17	≤12
Aminoglycosides	30μg	Neomycin(NEO)	≥17	13~16	≤12
	10 μg	Streptomycin(STR)	≥15	12~14	≤11
	10 μg	Gentamicin(GEN)	≥15	13~14	≤12
	30μg	Kanamycin(KAN)	≥18	14~17	≤13
Coumarins	30μg	Novobiocin(NOV)	≥17	13~16	≤12
Quinolones	30 μg	Enrofloxacin(ENR)	≥23	17~22	≤16
	5μg	Ciprofloxacin(CLX)	≥21	16~20	≤15
	5μg	Oxofloxacin(OFX)	≥16	13~15	≤12
	10μg	Norfloxacin(NOR)	≥15	13~16	≤12
	30 μg	Flumequine(FLU)	≥23	17~22	≤16
Sulfonamides	25μg	Sulfamisoxazole(SIZ)	≥17	13~16	≤12
	25μg	Trimethoprim/sulfamethoxazole(SMZ-TMP)	≥16	11~15	≤10
	5	Trimethoprim(TMP)	≥16	11~15	≤10
Macrolides	15	Erythromycin(ERY)	≥23	14~22	≤13
Rifamycins	5	Rifampin(RFP)	≥20	17~19	≤16
Polypeptides	10	Polymyxin B(PMB)	≥12	9~11	≤8
Nitrofurans	300	Furantoin(NFT)	≥17	15~16	≤14
	100	Furazolidone(FZD)	≥17	15~16	≤14
