# Supplementary material for: Antibiotic Resistance and Genetic Profiles of Vibrio parahaemolyticus Isolated from Farmed Pacific White Shrimp (Litopenaeus vannamei) in Ningde Regions
Source: Microorganisms. 2024 Jan 12;12(1):152. doi: 10.3390/microorganisms12010152 (PMC10821069; doi:10.3390/microorganisms12010152)
Supplement: Supplementary file 1 [file microorganisms-12-00152-s001.zip › Table S2.pdf]

Table S2. Primers and PCR conditions used in this study.

Function	Gene targets	Sequence of Primers (5'-3')	Annealing Temperature (°C)	References
Bacterial identification	16s rRNA	TACGGGAGGCAGCAG<br>AGGGTTGCGCTCGTT	55	10
Virulence genes identification	*tlh*	AAAGCGGATTATGCAGAAGCACTG<br>GCTACTTTCTAGCATTTTCTCTGC	55	10
	tdh	CCATCTGTCCCTTTTCCTGCC'<br>CCACTACCACTCTCATATGC	55	10
	trh	GGCTCAAAATGGTTAAGCG<br>CATTTCCGCTCTCATATGC	55	10
Antimicrobial resistance detection	*qnr VC136*	TTCTCACATCAGGACTTGC<br>GGAACAATGATTACCCCT	55	11
	qnr VC457	ATAAAACAGACCAGTTATATGT<br>ACTATTAAACVCTAATTGCTCTA	55	11
	qnr A	ATTTCTCACGCCAGGATTTG<br>GATCGGCAAAGGTTAGGTCA	55	11
	tet A	TTTCGGGGTTCGGGGATGGT<br>CAGGCAGAGCAAGTAGAGG	55	11
	tet M	GTGGACAAAGGTACAACGAG<br>CGGTAAAGTTCGTCACACACAC	55	11
	tet B	GTCGCGCATCGGTCAT<br>TTTTTCGCCCCATTTAGTG	55	11
	sul I	GTGACGGTGTTCGGCATTCT<br>TCCGAGAAGGTGATTGCGCT	63	11
	sul II	GCGCAGGCGCGTAAGCTGAT<br>CGAAGCGCAGCCGCAATTC	65	11

sul III	GCAACAGTTGGTGCTAAACGAGA<br>AGCAGATGTGATTGATTTGGGAG	56	11
str A	TGGCAGGAGGAACAGGAGG<br>AGGTCGATCAGACCCGTGC	54	11
str B	ATCGTCAAGGGGATTGAAACC<br>GGATCGTAGAACATATTGGC	52	11
erm	CCCGAAAAATACGCAAAATTTCAT<br>CCCTGTTTACCCATTTATAAACG	55	11
cat	ACAACAGCAACGGTACTAGC<br>CAACTTTCACCGATGCCAC	52	12
optr A	TTCTCACCCAGATATGCC<br>CGGGATCCCGGCAAACT	60	11
flo R	CTGCTGATGGCTCCTTTC<br>GCCGTGGCGTAACAGAT	57	11
cfr	GTGAAGCTCTAGCCAACCGTC<br>GCAGCGTCAATATCAATCCC	55	11
bla CARB	GCTGAGAGCTCATGAAAAAGTTA<br>CGTAGGATCCTTAACTTTATTTGTAGTGC	55	13

References

10. Han, H.; Li, F.; Yan, W.; Guo, Y.; Li, N.; Liu, X.; Zhu, J.; Xu, J.; Chen, Y.; Li, X.; Lv, H.; Zhang, Y.; Cai, T.; Chen, Y. Temporal and Spatial Variation in the Abundance of Total and Pathogenic Vibrio parahaemolyticus in Shellfish in China. *PLoS One*. **2015**,10(6):e0130302. doi: 10.1371/journal.pone.0130302.
11. Zhang, Z.; Lou, Y.; Du, S.; Xiao, L.; Niu, B.; Pan, Y.; Zhao, Y. Prevalence of Vibrio parahaemolyticus in seafood products from hypermarkets in Shanghai. *Journal of the Science of Food and Agriculture*. **2017**,97(2):705-710. doi: 10.1002/jsfa.7715.
12. Li , C.P.; Zhai, Q.Q.; Wang, X., Li, J. Isolation and Identification of *Vibrio parahaemolyticus* from Shrimp Culture Ponds and Analysis of its Drug Resistance and Virulence Genes. *Fisheries Science Progress.* **2020**, 9 (6) , 174-180.
13. Wang, J.; Zhan, Y.; Sun, H.; Fu, X.; Kong, Q.; Zhu, C.; Mou, H. Regulation of Virulence Factors Expression During the Intestinal Colonization of Vibrio parahaemolyticus. *Foodborne Pathogens and Disease*. **2022**,19(3):169-178. doi: 10.1089/fpd.2021.0057.
